# Maternal Characteristics and Socio-Economic Factors as Determinants of Low Birth Weight in Indonesia: Analysis of 2017 Indonesian Demographic and Health Survey (IDHS)

**DOI:** 10.3390/ijerph192113892

**Published:** 2022-10-26

**Authors:** Fatqiatul Wulandari, Trias Mahmudiono, Mahmud Aditya Rifqi, Siti Helmyati, Mira Dewi, Cindra Tri Yuniar

**Affiliations:** 1 Department of Nutrition, Faculty of Public Health, Universitas Airlangga, Surabaya 60115, Indonesia; 2 Department of Health Nutrition, Faculty of Medicine, Public Health, and Nursing, Universitas Gadjah Mada, Yogyakarta 55281, Indonesia; 3 Department of Community Nutrition, Faculty of Human Ecology, IPB University, Bogor 16680, Indonesia; 4 Department of Pharmacology and Clinical Pharmacy, School of Pharmacy, Institut Teknologi Bandung, Bandung 40132, Indonesia

**Keywords:** low birth weight, maternal characteristics, socio-economic factors, 2017 IDHS

## Abstract

Although low birth weight (LBW) is still a major health problem in Indonesia, studies about determinants of LBW with large sample sizes are still limited. This study aimed to examine the association between LBW and maternal characteristics, as well as socio-economic backgrounds in Indonesia. A secondary analysis of 2017 Indonesian Demographic and Health Survey (IDHS) was conducted, specifically using the questionnaires for women of childbearing age. A bivariate chi-square analysis and a multivariate logistic regression analysis were performed. As many as 6.7% of infants were born with LBW. In terms of maternal characteristics, women who gave birth to twins were 20.30 times more likely to have infants with LBW (*p* < 0.001). Women with birth intervals of <24 and ≥24 months were likely to have LBW infants (*p* < 0.05). Complications during pregnancy were also associated with LBW (1.99 times higher than women with no complications) (*p* < 0.001). In terms of socio-economic factors, women with higher education and higher wealth index were less likely to give birth to infants with LBW. Additionally, marital status and type of residence were also significantly associated with a higher risk of LBW. In conclusion, LBW was associated with maternal characteristics and socio-economic backgrounds among women of childbearing age in Indonesia, including twin births, birth interval, and pregnancy complications as well as educational attainment, wealth index, marital status, and type of residence.

## 1. Introduction

Low birth weight (LBW) is a serious public health problem and is considered to be one of the most important predictors of infant mortality in the first month of an infant’s life [1]. In 2015, the world’s largest prevalence of LBW was in the Asian region (17.3%), while LBW in the Southeast Asian region reached 7–21%. Indonesia was in the range of 5–<10% and ranked sixth among countries in Southeast Asia for LBW incidences [2,3]. The recent data from the 2018 Indonesian Basic Health Research Survey stated that Indonesia has a prevalence of 6% of LBW cases [4].

LBW is one of the health problems where prevention is very important because it has adverse short- and long-term effects. An estimated 1.1 million infants die from LBW complications each year [5]. In Indonesia, the neonatal mortality rate (NMR) reached 60,000 in 2019. This number has put Indonesia in the top 10 countries with the highest NMR in the world [6]. Besides its association with infant mortality, LBW is also tied to the inhibition of growth and cognitive development, as well as the increased risk of chronic diseases, such as cardiovascular disease and diabetes later in life [7].

LBW is generally recognized as a multifactorial health problem among infants, which includes both direct and indirect factors [8,9]. For direct factors, intrauterine growth retardation (IUGR), small for gestational age (SGA), or a combination of both are well known as the main causes of LBW [2]. However, in many cases, the causes of LBW remain unknown; they may include high maternal blood pressure, multiple births, acute infections, and other women’s pregnancy problems that can be categorized as maternal characteristics. In recent decades, with many socio-economic factors affecting health, not only have maternal characteristics affected LBW but socio-economic background have been considered as confounding factors in this problem, especially in developing countries [10]. The relationship between poor socio-economic background and LBW has often been attributed to poor maternal nutrition, especially during pregnancy, high prevalence of specific and non-specific infections, and birth complications underpinned by poverty [11].

In recent years, data from the household survey has become much more widely available and can be utilized to review the risk factors of LBW. Even though LBW is a major health issue in Indonesia, studies about LBW and its determinants using large sample sizes are still limited. Only three recent studies specifically examined the determinants of LBW with a large sample size. These studies were an undergraduate thesis using the 2017 Indonesian Demographic and Health Survey (IDHS), an article about determinant of LBW using the 2012 IDHS, and a conference proceeding using Indonesia’s Family and Health Survey 5 [12,13,14]. This current study utilized the data from the 2017 IDHS to examine maternal characteristics and socio-economic factors with a large sample size in order to gain insight into effective and efficient interventions that could be implemented in the future.

## 2. Materials and Methods

### 2.1. Data Sources and Study Design

Data were accessed from the latest 2017 Indonesian Demographic of Health Survey (IDHS) conducted by the Indonesian Central Statistics Agency, the National Population and Family Planning Agency, and the Indonesian Ministry of Health. This survey was financed by the Indonesian government from the allocated International Inner-City Fund (ICF) through the Demographic and Health Survey (DHS) program. DHS is a program run by the United States Agency for International Development (USAID), which provides funds and technical assistance for the implementation of population and health surveys in many countries. To be more specific, IDHS aims to monitor and evaluate health programs in Indonesia. The 2017 IDHS used four types of questionnaires regarding households, woman of childbearing age, married men (aged 15–49 years), and unmarried adolescents (aged 15–24) [15].

Households from 34 provinces in Indonesia were used as the population of this study. This survey was conducted nationally in rural and urban areas. A total of 49,261 households were selected in the survey, from which 48,216 households were located, and 99.5% (*n* = 47,963) of these households were successfully interviewed. From the interviewed households, there were 50,730 women of childbearing age interviewed, and 49,627 of them responded for a response rate of 97.8% [15].

The samples in the survey were taken from rural and urban areas, covering 1970 census blocks, of 49,250 households, consisting of 14,193 married men (aged 15–54 years), 24,625 unmarried male adolescents (aged 15–24 years), and 59,100 women of childbearing age (aged 15–49 years). The survey distinguished sample frameworks into two: household selection and sample census block selection [15].

A cross-sectional study design was used in this study. The research population from 2017 IDHS were used as samples for data cleaning. The study samples were women who were interviewed and had an infant born 5 years before the survey and had records of their infants’ birth weights, either from a written record or a women’s report. The total samples included 17,848 respondents from the children’s records dataset (IDKR71SV). IDKR71SV was the specific name with “KR” representing “kid’s records” and with “SV” representing code that the dataset could be statistically analyzed with by using SPSS (Statistical Package for the Social Sciences (New York, United States)). The samples were selected based on inclusion and exclusion criteria. The inclusion criteria were infants born with a recorded birth weight in 2017 IDHS and born alive or dead within 5 years before the survey. Respondents who did not complete the questionnaire, were missing data, or answered “do not know” were excluded from the final data cleaning. This study had been approved by the health research ethics committee from the Indonesian Ministry of Health as an ethical exemption, meaning there were no conflicts of interest.

### 2.2. Measures

Infants’ birth weight was the outcome studied. This variable was categorized into dichotomous data with “low birth weight” coded as “0” and “normal weight” coded as “1”. The WHO (2004) defines low birth weight as less than 2500 g at birth [5].

The independent variables used in this study were grouped into maternal characteristics and socio-economic factors. One of the maternal characteristics was maternal age at labor ranging from >35, <20, and 20–35 years. Parity was another characteristic, split into three categories: >4 children, 2–4 children, and 1 child (singleton). Gender of babies was divided into male and female. Twin births and pregnancy complications were both classified as “yes” and “no” options if these events occurred or not. Birth gaps were grouped into <24 months, 1st child, and ≥24 months.

As for socio-economic factors, marital status was labeled as never married or never in a formal relationship with their partners, married, living with partner or husband, widowed, divorced, and not living together. Desire for pregnancy was sorted into wanted then, wanted later, and wanted no more. Mother’s education level was classified as no education, incomplete primary, complete primary, incomplete secondary, complete secondary, and higher. Women’s occupations were classified into not working and has not worked in the last 12 months, professional, technical, managers and administration, clerical, sales, service, agricultural worker, industrial worker, and others. Residential location was grouped as either rural or urban. Wealth index was ranked as extremely poor, poor, middle, rich, and extremely rich. Wealth index was obtained by scoring each household’s assets based on the number and type of goods owned, ranging from televisions, bicycles, cars, and housing characteristics such as drinking water sources, latrine facilities, and main building materials. This score was calculated using a principal component analysis. The national wealth quintile was based on the total household score for each household member [15]. These data were obtained through questionnaires provided by the DHS program.

### 2.3. Analysis

Statistical Package for the Social Sciences (SPSS) 20 software was used to analyze the data in two steps. First, a bivariate analysis was used to analyze dependent and independent variables. Chi-square tests of independent variables were done to examine the association between each of the categorical independent variables and LBW, with an α-value of 0.05. Second, a multivariate analysis was done to determine how much influence the independent variables had on the dependent variables by using binary logistic regression, with odds ratios and 95% CIs.

## 3. Results

A total of 14,918 samples were selected after cleaning the data. Mean birth weight from infants was 3677 g ranging from 200 g up to a maximum of 9002 g. As many as 1236 infants were not weighed at birth. Table 1 reports the characteristics of 14,918 Indonesian women of childbearing age aged 15–49 years with infants who were born within 5 years before the survey, along with the bivariate analysis of maternal characteristics and socio-economic factors with LBW. The results show that 6.7% of women gave birth to infants with LBW, and more than fifty percent of all infants were male (51.6%). Most women gave birth between the ages of 20 and 35 years (76.8%). As many as 99.2% of infants were not twins. About two-thirds of all mothers had birth intervals of more than or equal to 24 months. As many as 17.4% or women had pregnancy complication although no specific complications were stated in the data.

As for socio-economic factors, most women were married (95.5%) and willing to have babies (83.3%), with more than one-fourth of the women having had completed secondary school. Women’s occupations were varied: nearly half of the women were not working and had not worked in the last 12 months. Interestingly, women from the extremely poor wealth status were the largest group at one-fourth of the sample, which was more than 5% higher than any other wealth index status. Additionally, type of residence was equally divided into rural and urban areas.

Table 1 also displays bivariate relationships between all independent variables and LBW status. In the maternal characteristics group, women aged below 20 years had the highest LBW rate (8.9%), followed by women aged above 35 years (6.8%) and 20 to 35 years (6.6%). Male infants were likely less to have LBW (6.4%) compared to female infants (7.1%) although the *p*-value for this factor was not significant. Women who had one child, or singleton birth, were highest in LBW at rates of 7.6%. Although there were no specific complications found, women who had any complications were likely to have infants with LBW (10.8%) at a rate 4.8% higher than those who did not have any complications. Twin infants were 9.4 times more likely to have LBW (59.3%) than non-twins.

As for socio-economic factors, women who had reported no longer living together had the highest percentage of LBW (10.2%) than any of the other four marital statuses. There were no substantial differences of the numbers of LBW among the three types of desire of wanted last child. At the educational level, women with no education were more likely to have LBW infants (10.2%). The number of LBW infants by women’s occupation varied, ranging from 5.6% up to 7.7% without substantial differences. Women from an extremely poor family were more significantly to have LBW infants (8.1%) compared to the four other classes of wealth. There were only slightly different LBW numbers among women who lived in rural or urban areas.

The multivariate results are displayed in Table 2. There were three significant variables in maternal characteristics. Women who gave birth to twins were 20.30 times more likely to have infants with LBW than those who did not (*p* < 0.001; OR = 20.30; 95% CI = 13.16–31.32). Moreover, women who experienced complications during pregnancy were also 1.99 times more likely to have infants with LBW than women without complications (*p* < 0.001; OR = 1.99; 95% CI = 1.72–2.31). Women with birth interval of <24 or ≥24 months had LBW infants significantly (*p* < 0.05; OR = 1.00 and OR = 2.06, respectively) compared to women who only had one child.

In socio-economic backgrounds, women who had higher wealth and education were significantly less likely to have infants with LBW. Marital status and type of residence were also identified as predictors of LBW. Women who were never married or never in a formal relationship with their partners were significantly more likely to have infants with LBW rather than women with any other marital status. Women who lived in urban areas were 1.18 times more likely to have LBW infants than women who lived in rural areas.

## 4. Discussion

This paper examined maternal characteristics as etiologic determinants of LBW and socio-economic factors as confounding factors of LBW based on the analysis of 2017 IDHS. Low birth weight as a multifactorial health problem is a significant contributor to mortality and morbidity among infants in developing countries. Other studies and reports show that LBW is a result of risk factors such as maternal characteristics [2,10,16,17,18]. Moreover, socio-economic factors could also be indirectly associated with LBW as confounding factors [19].

### 4.1. The Association between Maternal Characteristics and Low Birth Weight

Maternal characteristics were found to be associated with LBW. Women who had twins were at a higher risk of having infants with LBW than those who had singleton births. This result was similar to findings from a cross-sectional study in Nigeria, which reported that the risk of LBW of twin infants was significantly higher (*p* < 0.001) [20]. A study on 12,565 live-born Israeli twins indicated that this could happen because of the adaptive growth restrictions as a pattern of birth weight discordance in twin gestation [21]. In Indonesia, the twin birth rate was 14 per 1000 births in 2012, increasing from previous IDHS studies reporting 7.2 twin births per 1000 births in 1997–2007. This number could increase in the following years [22,23]. Demographic and Health Survey (DHS) data 1990–2000 also stated that in developing countries the risk of death from birth twins in the neonatal period is six times compared to that of single births [24]. This study suggests women with a familial history of twins should pay attention to their health during pregnancy by conducting regular antenatal care (ANC) visits.

Maternal complications were also significantly associated with LBW. Complications of pregnancy are health problems that occur during pregnancy. Pregnancy complications need to be avoided, as complications are collectively one of the important factors. Complications during pregnancy can involve women’s health, infants’ health, or both. Some pregnant women have problems that appear during pregnancy, and other women have problems even before they become pregnant, which thus causes complications. There are various kinds of complications that can be experienced by pregnant women, such as gestational diabetes mellitus (GDM), infection following birth, preeclampsia or (pregnancy-induced hypertension), vaginal bleeding and antepartum hemorrhage [25,26]. In this study, women who had complications were at a higher risk of giving birth to LBW infants, but a limitation of the 2017 IDHS is that complications were not specifically stated. Nevertheless, this finding was significantly supported by both bivariate and multivariate analysis; therefore, the variable of pregnancy complications can be considered as a risk factor for LBW. This result supports previous studies in Qatar and India: those studies associated maternal complications such as gestational diabetes mellitus, antepartum hemorrhage, and pregnancy-induced hypertension with LBW [18,27]. It is very important for pregnant women to receive antenatal care before and during pregnancy to reduce the risk of pregnancy complications.

Birth intervals were also significantly associated with LBW. Women who had <24 months or ≥24 months birth interval were likely higher of having LBW infants. A study from 2013 through 2018 of women enrolled in the NICHD Global Network Maternal Newborn Health Registry (MNHR) in six low and lower-middle income countries also supported these findings. Women with both short and long birth intervals had increased risk of adverse maternal and neonatal outcomes, although the birth intervals were varied among these countries [28]. Another study reported that a short interpregnancy interval (<24 months) was more likely with a higher risk of LBW [29]. Family planning programs to extend the birth interval to a minimum of two years would, therefore, help to decrease LBW [10].

In contrast, this study did not find women’s age at childbirth, parity, or sex of child to be significant in the regression analysis, whereas bivariate results found the opposite for women’s age and parity. Socio-economic backgrounds might be confounding factors for maternal characteristics. Women with higher education and wealth were less likely to have LBW infants, although women of lower age or with singleton infants (women aged <20 years with 8.9% LBW and singleton infant with 7.6% LBW) were not significantly associated with LBW). A study from Chicago households enrolled in the Study of Household Purchasing Patterns, Eating, and Recreation [SHOPPER] stated that higher income households, adjusted with education, marital status, age, and race, purchase more healthy foods compared to lower income households. Food purchasing patterns may mediate income differences in dietary intake quality [30]. Healthy diet before and during pregnancy can protect women from undernutrition and/or infection. Moreover, it can be possible that lower maternal age and parity still had associations with LBW even though they did not significantly increase LBW.

### 4.2. The Association between Socio-Economic and Low Birth Weight

As hypothesized, this study found an association between socio-economic factors and LBW. In line with other studies, women with higher education and wealth were less likely to give birth to infants with LBW. These findings are reinforced by the significant values obtained through the chi-square analysis. A study in Malawi indicated that LBW infants were more commonly given birth to by women with no education and lower wealth status [31]. A meta-analysis from the MEDLINE database that examined the relationship between maternal education levels and LBW also discovered the same results [22]. A study in Lombok, Indonesia also found that social status (28%), lower levels of education (5%), and non-modifiable factors such as wealth index contributed to the risk of LBW (17%) [32]. In Indonesia, 28% of the population were families at the lowest wealth index. It was also found that 30% of Indonesian women aged 15–49 years did not finish high school [15].

Marital status and type of residence were also significantly associated with increased risk of LBW. Women who never married or never in a formal relationship with their partners were significantly more likely to give birth to LBW infants than married women. This result was similar to that of a study in the United States that found that women who reported being unmarried and having no paternity status were 1.6 and 1.4 times more likely to have preterm low birth weight and term low birth weight infants, respectively [33]. In contrast to other studies, this study found that LBW infants were more commonly born in urban areas. This finding was different from the results of the Cambodian Demographic and Health Survey in 2010–2014, which stated that LBW was higher in rural areas (8%) in comparison with urban areas (6%) [34]. Documented evidence has shown that rural women were less sedentary and participated in more household/caregiving activities rather than occupational activities than urban women [35]. Another factor from rural areas that could suppress the reported number of LBW is that of rural households storing more food for future consumption than urban households. This could impact the nutritional status of pregnant women [36]. Meanwhile, there were substantial disadvantages to living in urban areas compared to rural areas due to social or environmental factors. In rural areas, lower air pollution exposure and higher rates of working women had significant protective or opposing relationships with LBW [33,35,36].

This study’s main limitation was missing data in the dataset. However, this study still had a large sample size and provided strong evidence of determinants of low birth weight in Indonesia, including several variables grouped into two classes: maternal characteristics and socio-economic factors. In the future, especially regarding the upcoming Indonesian Demographic and Health Survey, this five-year survey should be conducted carefully to maintain completeness of the data so that abundant missing data can be prevented. More studies related to low birth weight, using more specific and detailed data, are needed to create an effective and efficient healthcare interventions in the future.

## 5. Conclusions

In conclusion, LBW was associated with twin births, birth interval, and pregnancy complications, as well as socio-economic factors including educational background, wealth index, marital status, and type of residence. Based on these findings, policy makers and government should place an emphasis on the quality and services of antenatal care (ANC) for pregnant women so that complications can be prevented as well asbirth intervals and twin births can be better mitigated by healthcare providers. Moreover, this study also recommends that women with lower educational and wealth index backgrounds be offered free ANC services to improve their health quality during pregnancy. Lower wealth index and education status were two confounding factors that could have a substantial impact on LBW. Adequate antenatal care-based interventions, especially on women with lower socio-economic backgrounds, are expected to suppress the rate of LBW.

## Figures and Tables

**Table 1:** Characteristics of 14,918 women aged 15–49 years with infants born within five years before 2017 IDHS and bivariate analysis of maternal characteristics and socio-economic factors with LBW.

Variables	*n*	%	*n* (%)	df	*p*-Value
**Dependent Variables**					
Weight of child					
Normal weight	13,912	93.3	-	-	-
Low birth weight	1006	6.7	-	-	-
**Independent variables**					
**Maternal characteristics**					
Women’s age at childbirth					
>35	2553	17.1	174 (6.8)	2	0.023
<20	907	6,1	81 (8.9)		
20–35	11,458	76,8	751 (6.6)		
Parity					
>4 children	1066	7.1	73 (6.8)	2	0.01
2–4 children	9195	61.6	577 (6.3)		
Singleton	4657	31.2	356 (7.6)		
Sex of child					
Male	7694	51.6	491 (6.4)	1	0.071
Female	7224	48.4	515 (7.1)		
Child is twin					
No	14,800	99.2	936 (6.3)	1	<0.001
Yes	118	0.8	70 (59.3)		
Preceding birth interval (months)					
<24 months	969	6.5	71 (7.3)	2	<0.001
1st child	4684	31.4	376 (8.0)		
≥24 months	9265	62.1	559 (6.0)		
Complications during pregnancy					
No	12,325	82.6	726 (5.9)	1	<0.001
Yes	2593	17.4	280 (10.8)		
**Socio-economic factors**					
Marital status					
Never married	23	0.2	5 (21.7)	4	0.002
Married (living with partner)	14,447	96.8	957 (8.1)		
Widowed	88	0.6	8 (9.1)		
Divorced	301	2.0	30 (10.0)		
No longer living together/separated	59	0.4	6 (10.2)		
Wanted last child					
Wanted then	12,434	83.3	854 (6.9)	2	0.283
Wanted later	1326	8.9	76 (5.7)		
Wanted no more	1158	7.8	1006 (6.7)		
Women’s level of education					
No education	157	1.1	16 (10.2)	5	<0.001
Incomplete primary	999	6.7	78 (7.8)		
Complete primary	2687	18.0	219 (8.2)		
Incomplete secondary	3862	25.9	255 (6.6)		
Complete secondary	4586	30.7	298 (6.5)		
Higher	2627	17.6	140 (5.3)		
Women’s occupation					
Not working and has not worked in the last 12 months	6921	46.4	490 (7.1)	8	0.233
Professional, technical	1208	8.1	68 (5.6)		
Managers and administration	81	0.5	6 (7.4)		
Clerical	663	4.4	29 (4.4)		
Sales	2548	17.1	173 (6.8)		
Service	982	6.6	65 (6.6)		
Agricultural worker	1622	10.9	108 (6.7)		
Industrial worker	880	5.9	66 (7.5)		
Other	13	0.1	1 (7.7)		
Wealth index					
Extremely poor	3794	25.4	306 (8.1)	4	0.001
Poor	2969	19.9	207 (7.0)		
Middle	2846	19.1	183 (6.4)		
Rich	2734	18.3	172 (6.3)		
Extremely rich	2575	17.3	138 (5.4)		
Type of residence					
Rural	7484	50.2	502 (6.7)	1	0.861
Urban	7434	49.8	504 (6.8)		

*n* = number of each variable; *n* (%) = number and percentage of BBLR-related sample within each variable; df = degrees of freedom; α = 0.05.

**Table 2:** Multivariate analysis of maternal characteristics and socio-economic factors with LBW among women of childbearing age.

Variables	OR	95% CI
**Maternal characteristics**		
**Women’s age at childbirth**		
**>35**	1.00	[1.00–1.00]
**<20**	0.95	[0.68–1.32]
**20–35**	0.88	[0.72–1.07]
**Parity**		
**>4 children**	1.00	[1.00–1.00]
**2–4 children**	1.18	[0.88–1.57]
**Singleton**	0.61	[0.22–1.72]
**Sex of child**		
**Male**	1.00	[1.00–1.00]
**Female**	1.13	[1.99–1.29]
**Child is twin**		
**No**	1.00	[1.00–1.00]
**Yes**	20.30 ***	[13.16–31.32]
**Preceding birth interval (months)**		
**<24 months**	1.00 *	[1.00–1.00]
**1st child**	0.75	[0.57–0.98]
**≥24 months**	2.06 *	[0.75–5.65]
**Complications during pregnancy**		
**No**	1.00	[1.00–1.00]
**Yes**	1.99 ***	[1.72–2.31]
**Socio-economic factors**		
**Marital status**		
**Never married**	1.00 *	[1.00–1.00]
**Married (living with partner)**	0.37	[0.12–1.13]
**Widowed**	0.42	[0.12–1.45]
**Divorced**	0.39	[0.13–1.15]
**No longer living together/separated**	0.42	[0.11–1.55]
** Wanted last child**		
**Wanted then**	1.00	[1.00–1.00]
**Wanted later**	0.78	[0.61–1.01]
**Wanted no more**	0.96	0.74–1.25
**Women’s level of education**		
**No education**	1.00 *	[1.00–1.00]
**Incomplete primary**	0.71	[0.39–1.29]
**Complete primary**	0.79	[0.45–1.41]
**Incomplete secondary**	0.62	[0.35–1.09]
**Complete secondary**	0.60	[0.34–1.07]
**Higher**	0.52 *	[0.28–0.96]
**Women’s occupation**		
**Not working and has not worked in the last 12 months**	1.00	[1.00–1.00]
**Professional, technical**	0.99	[0.72–1.36]
**Managers and administration**	1.34	[0.56–3.21]
**Clerical**	0.14	[4.88–1.12]
**Sales**	0.98	[0.81–1.18]
**Service**	0.87	[0.66–1.16]
**Agricultural worker**	0.87	[0.69–1.09]
**Industrial worker**	1.03	[0.78–1.36]
**Other**	1.34	[0.17–10.47]
**Wealth index**		
**Extremely poor**	1.00 *	[1.00–1.00]
**Poor**	0.86	[0.71–1.05]
**Middle**	0.76 *	[0.62–0.95]
**Rich**	0.73 **	[0.58–0.92]
**Extremely rich**	0.64 **	[0.49–0.83]
**Type of residence**		
**Rural**	1.00	[1.00–1.00]
**Urban**	1.18 *	[1.02–1.38]

Statistical test using binary logistic regression; * *p* < 0.05; ** *p* < 0.01; *** *p* < 0.001; OR = Odds Ratio; CI = Confident Interval.

## Data Availability

Data were withdrawn from the website of Indonesian Demographic and Health Survey (IDHS).
